# Exploring the Utilisation of Stand up Paddle Boarding in Australia

**DOI:** 10.3390/sports5030053

**Published:** 2017-07-22

**Authors:** Ben Schram, James Furness

**Affiliations:** Water Based Research Unit, Bond Institute of Health & Sport, Faculty of Health Sciences and Medicine, Bond University, Gold Coast, QLD 4226, Australia; jfurness@bond.edu.au

**Keywords:** water sports, stand up paddle, paddle boarding, SUP

## Abstract

Stand Up Paddle Boarding (SUP) has grown exponentially in the last few years with unprecedented participation rates globally. Despite some scientific research on physiological and performance variables, minimal information exists regarding participation and utilisation. The purpose of this study was to discover more about how and where people participate in the relatively new sport of SUP. An open-source online survey application was administered internationally to active SUP participants to capture information relevant to both demographics and participation. Of a total of 240 responses, 154 (64.2%) were Australian. The average SUP rider was 42.9 ± 11.7 years, mass 80.4 ± 18.7 kg, 1.75 ± 0.10 m tall with a BMI of 26.1 ± 4.9. More males (69.5%) participate in SUP than females with the majority of participants from the eastern seaboard of Australia. Participants most commonly used SUP for fun and fitness, for around 3 h per week, predominantly at the beach with friends, with around half of the respondents reporting a competitive involvement. This is the first study to date to quantify participation of SUP within Australia. Results revealed SUP is a global activity with a high representation within Australia. Key findings from this study reveal the geographical and demographic distribution of SUP use. Consequently, these findings may inform the industry about its target audience. Additionally, information regarding the ‘typical’ SUP rider may serve to further promote and grow the sport.

## 1. Introduction

The popularity of Stand Up Paddle Boarding (SUP) has seen rapid growth in the last few years both as a recreational activity and as a professional sport [1]. The activity involves paddling while standing, utilising a long single bladed paddle on a board larger and wider than a traditional surfboard [2,3]. SUP was originally used to take photos of tourists learning to surf in Hawaii post World War 2, however SUP’s popularity spiked late in the 2000’s once professional surfers and watermen were seen to utilise it as a training tool during periods of small surf [4].

**Figure 1:**
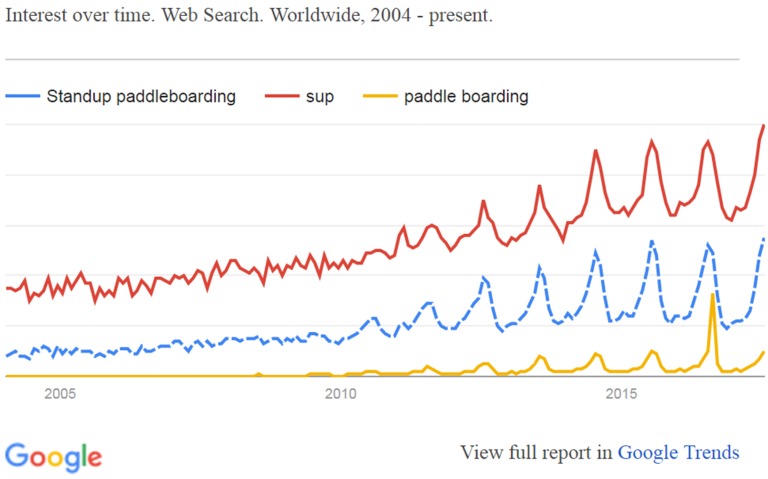
Google trends representation of the interest in SUP [8].

Despite minimal research in participation rates in scientific manuscripts, there are numerous reports available online regarding SUP’s popularity. In the year 2013, SUP had the most first time participants of any sport in the USA [5], and 1.2 Million people tried SUP in 2011, a number up 18% from the year before [6,7]. Retailers of kayaks estimated that 15–20% of their sales could be attributed to SUP in 2012 [6]. In addition, Figure 1 illustrates the Google Trends illustration of interest over time in SUP. The most commonly searched term is ‘SUP’, while other terms pertaining to SUP include ‘standup paddleboarding’ and ‘paddle boarding’.

Understanding participation rates in sport is vital to assist in both strategic planning and policy development of sport [9]. There are clear, positive associations between physical activity and physical and mental health [10,11,12]. Information regarding participation and utilisation of sporting activities informs sporting governing bodies, sports agencies, sport and recreation and health organisations as an aid to increase participation [13]. Additionally, it can also be useful from an industry perspective where-by data of this nature informs marketing and production decisions regarding extent and intensities of demand of products and activities [14]. 

Given the relatively new nature of SUP and the minimal information regarding the utilisation of the unique exercise modality, an investigation was conducted. The purpose of this study was to investigate participation rates of SUP using an online survey platform. Key information of interest included geographical location, duration of sessions, type of SUP activity, the level of instruction provided prior to commencement, group participation and competitive history.

## 2. Methods

A cross-sectional cohort study was designed utilising an online survey platform (Qualtrics, Provo, UT, USA). This study was granted research ethics approval by the University Human Research Ethics committee (RO 1540). 

There were three components to this survey including (1) introduction; (2) demographics and participation; (3) competitive history. Background information was contained in Section 1, demographic and participation questions were found in Section 2. Section 2 included questions regarding age, gender, and information as to how a participant was first introduced to SUP. Section 3 contained questions regarding SUP lessons and competitive involvement. 

Inclusion criteria involved participants who were active SUP riders with at least 12 months experience. The survey was available between January 19, 2016 and March 21, 2016, and was distributed to both local and international SUP organisations, including the international governing body ‘Stand Up Paddle Athletes Association (SUPAA)’. In addition, the survey was advertised via social media outlets including Facebook, Instagram, and Twitter, various radio spots, and through online newsletter features.

## 3. Statistical Analyses

The data was analysed using the Statistical Package for the Social Sciences (SPSS, version 23.0) [15]. Frequencies and descriptive statistics were used to summarise each variable. Independent *t*-tests were used to determine differences between each continuous variable. Statistical significance was set at *p* < 0.05 alpha priori. 

## 4. Results

A total of 309 data samples were initially extracted for data analysis. Sixty-nine participants (22.3%) either did not progress past the consent page or did not provide adequate information to be included in the data analysis and were excluded. As a result, 240 participant data sets were included in the analysis. Of the 240 entries, the majority of respondents were Australian (64.2%, *n* = 154) with the USA (21.7%, *n* = 52) making up much of the remaining respondents. Responses were also seen from the UK, New Zealand, Canada, Brazil, Fiji as well as Asia and Europe in general. As this study was designed to understand more about SUP participation in Australia, only the Australian data was included for the rest of this study. 

## 5. Demographics

**Figure 2:**
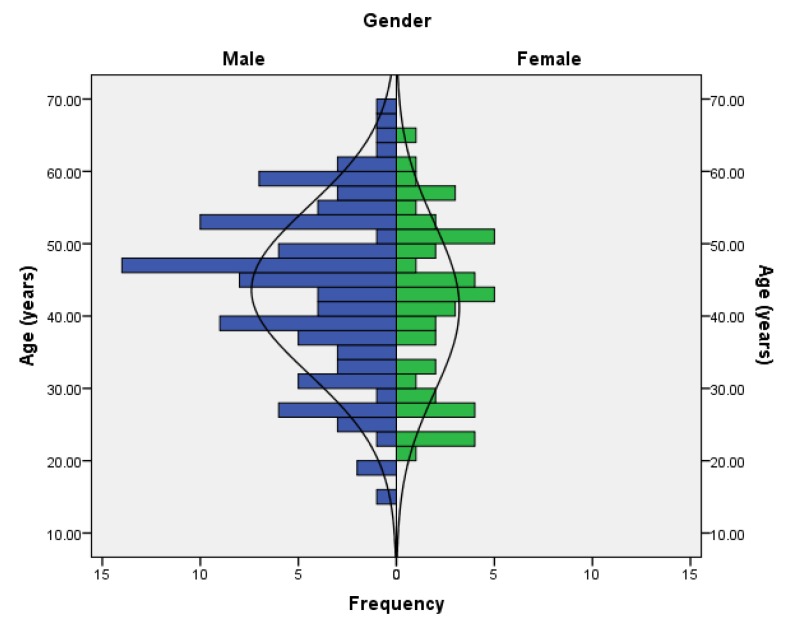
Age distribution of Australian SUP riders.

The respondent’s age, weight, height and BMI can be seen in Table 1. Males were on average older than the females, with the difference not being significant. Males were also significantly (*p* < 0.05) of greater mass (+28.1%) and taller (+6.6%) than the females, with a non-significantly higher BMI (+9.4%) than the females. The age distribution of respondents for both males and females can be seen in Figure 2. The range of males’ ages was 15 to 68 years and that of females was 20 to 68 years. 

**Figure 3:**
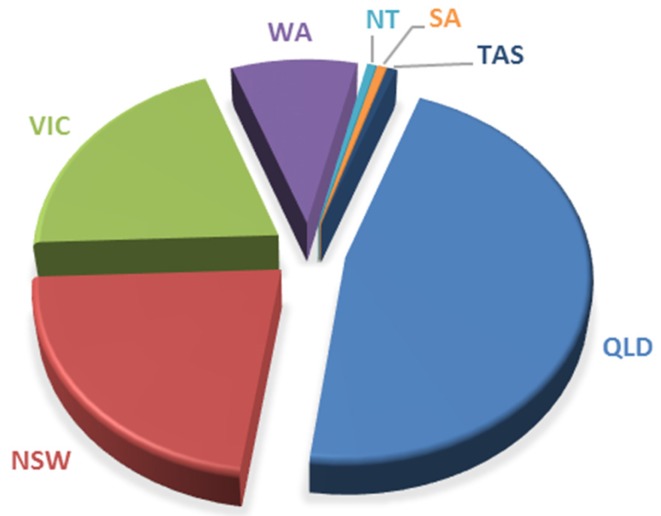
SUP participation by state.

Figure 3 represents the distribution of the Australian participants; 46.8% (*n* = 72) were from Queensland, 22.1% (*n* = 34) from New South Wales, 20.1% (*n* = 31) from Victoria, 9.1% (*n* = 14) from Western Australia and 0.6% (*n* = 1) from each of the Northern Territory, Tasmania and South Australia. When specifically looking at participation within Queensland, both Currumbin & Tallebudgera were hotspots, with 33.3% and 18% of participants, respectively. The Central Coast (9.7%) and Sydney (4.5%) were the most popular spots in New South Wales, while Port Phillip Bay (14.3%) had the most respondents in Victoria.

Respondents had been paddling an average of 3.8 ± 2.3 years, with males averaging 3.7 ± 2.4 years, significantly longer (*p* < 0.05) than females, with 2.8 ± 1.5 years. Males spent, on average, 4.5 ± 3.2 h per week doing SUP, with females spending 3.5 ± 3.3 h. Just over 38% of respondents reported that they had a personal interest in the sport which led them to begin SUP, while another 35.1% transitioned from another water-based sport. Introduction to the sport from a friend (22.1%) or family member (4.5%) were other ways through which people were introduced to SUP. The primary reason for using a SUP was for fun and fitness (44.8%) and surfing (36.4%), while other reasons included racing (16.9%) and touring (1.9%). 

Respondents were mostly using their SUP’s in a beach or bay (74%) or creek (13%), with few people utilising a river (9.7%), lake (1.9%), canal or dam (0.6%) or harbour (0.6%). Most commonly, people paddled with friends (31.8%), or alone (28.6%), or as part of a wider group in a SUP club (27.9%), with just over half (53.9%) having received instruction on how to paddle a SUP. Of the people who had received instruction, in only 39.6% of these cases was the person giving the instructions adequately qualified. 

Half of the surveyed population reported being involved with SUP in a competitive way, with the other half using their SUP recreationally. Of the competitive riders, most (37%) were competing locally, with 6% competing on a state level, and 6.5% competing on a national level. Only one respondent reported an international level competitive involvement. 

## 6. Discussion

The purpose of this study was to understand more about the way in which people utilise SUP as both a recreational activity and as a competitive sport within Australia. The results of this study suggest SUP is a global sport with participation recorded in North America, South America, Asia, Europe, Australia, New Zealand and the Pacific Islands. To our knowledge, this is the first study to highlight participation rates of the growing sport of SUP. 

Some differences were found in this study when comparing the results from previous reported figures in regard to gender participation. In this study, there was a higher number of male respondents than females (69.5% vs. 30.5%). Slightly higher participation rates of females have previously been reported, with 42% of participants in SUP being female [7]. The percentage of females in this study however was much greater than in surfing studies, where only 8.7–10% of respondents were female [16,17,18]. For females specifically, numerous government initiatives exist in Australia to become more active, such as ‘Coasting’ [19], which specifically uses SUP for both the documented health benefits and social involvement [20]. Considering the results of the current study and the increase in government initiatives promoting female-specific exercise programs, SUP could be an effective tool in engaging female participants. 

When comparing the hours in which people SUP, both males and females utilised SUP less than what surfers have reported. In a previous study, surfers spent an average of 6.7 ± 5.6 and 7.3 ± 6.8 hours per week surfing, respectively. This may be due to the intermittent nature of surfing, thought to consist of surfing a wave only 2.5% to 8% of the time [21,22], as opposed to the more continuous nature of SUP, where elevated heart rates can be maintained throughout the sessions [23]. Previous research shows that people who surf (both short and long boards), paddle outrigger canoes and surf life savers often use SUP for cross training [4], with many of Australia’s top paddlers being previously successful in both outrigger paddling and surf lifesaving. 

The findings from this study would suggest that there is a heavy representation of SUP riders on the eastern seaboard, mainly paddling in the oceans. The percentages found in this study appear to be in line with the number of SUP clubs registered with Surfing Australia, the representing body on the International Surfing Association. Currently, 57% of the registered SUP clubs are in Queensland, 21.4% in New South Wales and 7% in Victoria. Only one club is registered in both South Australia and Western Australia, with no registered Northern Territory clubs found. 

The fact that few people have received any instruction before paddling a SUP may warrant concern. A person being adequately qualified to instruct SUP in Australia is an Academy of Surfing Instructors (ASI) recognized instructor who has completed theory workshops, practical assessments, tests and first aid certificates. Without this level of instruction, a paddler may be more prone to injury. Injuries associated with SUP participation are thought to include the elbow, shoulder and back, and could be due to improper technique and inadequate training [4,24]. Given that the primary reason people use a SUP is for fun and fitness, SUP may be an ideal activity to promote to address the problem with inactivity amongst Australians. 

The high number of Australian respondents should be viewed with caution, as it was an Australian based survey. Despite being sent to the international body of SUP, the Stand Up Paddle Athletes Association (SUPAA), it would appear as though this may not be a true representation of current participation rates. When viewing the current top 100 competitive males and 50 females, Australia comprises only 12% of these individuals, with continental USA comprising 25%, Hawaii being 14% and a strong representation from France (9%), Tahiti (6%), Italy (5%) and New Zealand, Mexico and the UK (3% each). In addition, given that this study was implemented in Queensland, this may also have led to a higher involvement of local SUP paddlers. Given the small number of respondents, the results of this study are only applicable to this sample and may not be reflective of the SUP population in Australia as a whole. 

## 7. Conclusion 

This is the first paper to present demographic and participation data specific to SUP. SUP appears to be a global activity with a high representation in Australia and more specifically on the eastern seaboard. SUP appears to be used by a variety of different ages with both male and female utilisation. Equal numbers of people appear to use SUP for recreational and competitive purposes. Most people appear to use SUP’s for around 3 h per week, for fun and fitness at the beach, with the majority having never received any instructions on proper paddle technique. 

## Figures and Tables

**Table 1:** Demographic Information of SUP Participants.

Parameter	Total (*n* = 154)	Males (*n* = 107)	Female (*n* = 47)
Age (years)	42.9 ± 11.7	43.6 ± 11.6	41.2 ± 11.7
Mass (kg)	80.4 ± 18.7	88.1 ± 22.6 *	68.8 ± 16.6
Height (m)	1.75 ± 0.10	1.78 ± 0.07 *	1.67 ± 0.08
BMI (kg/m^2^)	26.1 ± 4.9	26.8 ± 4.8 *	24.5 ± 4.6

* significant difference (*p* < 0.05).
